# Niemann-Pick Disease: An Underdiagnosed Lysosomal Storage Disorder

**DOI:** 10.1155/2019/3108093

**Published:** 2019-04-21

**Authors:** Inusha Panigrahi, Manoj Dhanorkar, Renu Suthar, Chanchal Kumar, Mullai Baalaaji, Babu Ram Thapa, Jasvinder Kalra

**Affiliations:** ^1^Department of Pediatrics, PGIMER, Chandigarh, India; ^2^Department of Gastroenterology, PGIMER, Chandigarh, India; ^3^Department of Obstetrics and Gynecology, PGIMER, Chandigarh, India

## Abstract

Lysosomal storage disorders (LSDs) collectively constitute a significant public health burden in developing countries. Commoner LSDs include Gaucher, Fabry, and Niemann-Pick disease (NPD), but many cases remain undiagnosed. With the high incidence of consanguineous marriages, South East Asian countries are expected to have high prevalence of these LSDs. Here we report 4 cases of NPD type A/B in 3 families presenting with hepatosplenomegaly and cytopenias including one family with two sibs having hypertension and mitral valve prolapse. The diagnosis of NPD was proven by mutation analysis with identification of novel mutations, including a novel 4 bp insertion mutation (C>CCTGG) in exon 2 of the* SMPD1* gene. We also had two cases of NPD type C, confirmed on mutation analysis.

## 1. Introduction

High prevalence of LSDs has been reported from some South East Asian populations [1–5] and Saudi Arabia, possibly because of increased frequency of consanguineous matings. Niemann-Pick disease (NPD) is a lysosomal storage disorder which presents with hepatosplenomegaly, jaundice, and cytopenia. In the severe form of disease patient can present early with failure to thrive, hepatosplenomegaly, or pulmonary manifestations [6]. The clinical manifestations have been classified into type A, type B, and type C disease presentations. Mutations in types A and B are seen in SMPD1 gene, but in type C disease are seen in* NPC1 *and* NPC2* genes. Types A and B result from the deficient activity of sphingomyelinase and the* SMPD1* gene located on bands 11p15.1-p15.4 [7] and are referred to as acid sphingomyelinase deficiency (ASMD). The enzymatic defect results in pathologic accumulation of sphingomyelin, a ceramide phospholipid, and other lipids in the monocyte-macrophage system [8, 9]. Type A is most severe form of the disorder, resulting in early death [10]. In many cases the diagnosis rests on identification of cherry red spots in fundus on ophthalmic evaluation or presence of storage cells in the bone marrow [11]. The enzyme analysis is available only in selected laboratories for sphingomyelinase and may not be accessible in all suspected cases. For type C disease, the diagnosis traditionally required a liver biopsy and filipin staining. Nowadays, biomarker studies and molecular testing for* NPC1/C2 *genes have helped in better diagnosis. Small molecule therapy has been tried in NPD but further research is needed to devise better treatment modalities for the disorder.

This is a retrospective analysis of cases of Niemann-Pick disease detected on follow-up in Genetic Clinic, of a tertiary care centre. Earlier bone marrow evaluation was routinely done for diagnosis of storage disorder, of which commonest is Gaucher disease in patients presenting with hepatosplenomegaly and cytopenias. Nowadays with better expertise and availability of good laboratory facilities, sometimes enzyme analysis is done upfront for diagnosis of storage disorder.

## 2. Case Presentation

In retrospective analysis of children undergoing bone marrow evaluation for suspected storage disorder (Nov 1998-October 2008), we found oldest child of 12-year age (range: 1 month to 12 years). There were 21 were typical Gaucher cells having cytoplasm with crumpled/crinkled tissue paper appearance and 27 were diagnosed as non-Gaucher storage disorder. Four cases with ASMD-Niemann-Pick disease type A/B and 2 cases of Niemann-Pick disease type C are described below.

Enzyme analysis was done by fluorimetric assay using standard protocols and use of control enzyme for increasing reliability of the result. Normal value of acid sphingomyelinase taken was 10-32 nmol/hr/mg, and chitotriosidase normal level was 28.66-62.94 nmol/ml/hr. Mutation analysis was done by Sanger sequencing or by next generation sequencing (NGS) with targeted analysis for storage disorder genes. In Sanger sequencing, all the six exons were amplified and sequenced bidirectionally on ABI DNA Sequencer. For NGS-Clinical Exome test, 5000 genes were amplified using Illumina platforms, and target genes were analysed by standard bioinformatics databases. Consent for testing was taken from the parents/couple who came for genetic counseling or molecular testing.

In* family 1*, the 27-year-old female presented for antenatal counseling in the fetal medicine clinic. She had history of 2 previous children who had expired in early childhood with seizures and hepatosplenomegaly. The enzyme analysis done in the second child was available and was consistent with NPD. The prenatal diagnosis was done on CVS sample, which was borderline low (N range-72-210 nmol/hr/mg) and the parents decided to continue the pregnancy. Subsequent analysis of* SMPD1* gene was performed by Sanger sequencing. Two heterozygote mutations in the* SMPD1* gene were detected. One was the R542 X mutation resulting from C→T substitution at nucleotide position C1624 in exon 6 of* SMPD1*. The other mutation was C387F, resulting from G→T substitution at nucleotide position C1160 in exon 3 of* SMPD1*. The mother carried the C387F mutation and the father had the R542X mutation. The family was recalled and counseled accordingly. The database search showed two known mutations -R542X and mutation C387F or p.Cys287Phe, in* SMPD1*.

In* family 2*, the 5-month-old male child, resident of Muzaffarnagar, UP state, presented with episodic loose stools and progressive abdominal distension since birth. Child was born at term with birth weight of 2.25 kg and no history of neonatal encephalopathy. Child was on top feeds since birth. As the child was growing, parents started noticing episodic loose stools in child which was unrelated to the changes in the type of feeds. Parents also started noticing progressive abdominal distension onset since first month of life which was involving the whole abdomen. This was not associated with waxing and waning phenomenon, unrelated to feed and bowel habit, not in form of isolated palpable mass in abdominal quadrant. This time he presented with 15-day history of fever associated with respiratory distress and irritability for last 2 days prior to admission, no history suggestive of jaundice, high colored urine, clay colored stool, seizure, and bleeding manifestation. In developmental history, he was having mild delay in motor and cognition sectors. He was born to third degree consanguineous couple with no family history of similar illness. At presentation to the hospital he was having respiratory distress, otherwise hemodynamically stable. He was having coarse facies, grade II malnutrition according to IAP classification, and severe wasting according to WHO classification with small head. He also had firm hepatosplenomegaly, liver of 4 cm below right costal margin with span of 9 cm, firm in consistency, and being with palpable left lobe. Spleen was 3 cm along splenic axis below left costal margin. Child had right sided reducible inguinal. On central nervous system examination child was found to have generalized hypotonia. The possibilities considered were intrauterine infection/metabolic or storage disorder with acute presentation with community acquired pneumonia. Complete blood count was suggestive of microcytic and hypochromic anemia with normal platelets and leukocytosis. Biochemical parameters were suggestive of elevated transaminases-AST-229 and ALT-489 units. Toxoplasma and CMV serology were negative and HIV ELISA was nonreactive. The tubercular work-up, including Mantoux test, and 3 gastric aspirates for AFB were negative. Ultrasound abdomen showed hepatosplenomegaly with normal echotexture of liver and normal kidneys and cranium. Fundus examination showed bilateral cherry red spots. Radiological evaluation did not show any dysostosis in the spine and hand radiographs. Bone marrow examination revealed foamy vacuolated large sized macrophages with size of 25-30 times the size of mature lymphocytes or foam cells, consistent with storage disorder. As per the next step of investigation enzyme analysis was done for NPD which was suggestive of low sphingomyelinase level. Hence a diagnosis of Niemann-Pick disease likely type A was made in the index child. However, unfortunately child succumbed to an illness three months later at 8 months of age.

**Figure 1:**
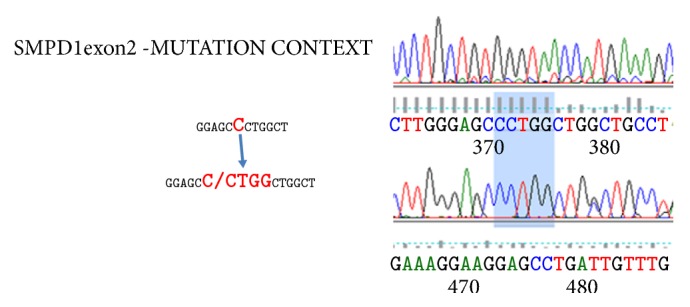
Representation of the novel 4 bp insertion mutation in SMPD1 gene in exon 2 in family 2 in parental sample.

The family was initially counseled regarding enzyme analysis on amniotic fluid. However, in the subsequent pregnancy, they did not come for prenatal diagnosis, and a girl child was born. She was also similarly affected and expired due to an illness at 4 month of age. The parents came to the genetics clinic again after death of the second child. DNA mutation analysis was done for* SMPD1* gene by Sanger sequencing, and both parents were heterozygous for the same 4 bp insertion mutation (C>CCTGG) in exon 2 of the gene (Figure 1).

In* family 3*, a 9-year-old male child, a case presenting with short stature and hepatosplenomegaly, with suspected NPD type B, homozygosity for a novel missense variant c.1566T>G in exon 6 of the* SMPD1 *gene, was NGS and confirmed by bidirectional Sanger sequencing. He had hepatosplenomegaly and recurrent respiratory problems. The male child was admitted in pediatric ICU for left focal motor seizures and also had features of acute on chronic malnutrition, facial dysmorphism (mild coarse facies; prominent ears), and large hepatosplenomegaly and hypertensive records at admission. Otherwise his neurological examination was essentially normal. MRI brain done was suggestive of T2 hyperintensities in left side of pons and medulla with patchy contrast enhancement. He also had hypertensive emergency during hospital stay with seizure, pulmonary edema, and grade 4 hypertensive changes in retinal vessels and nonoliguric acute kidney injury which responded to pharmacological management. He subsequently developed pancytopenia, coagulopathy, and sudden onset hypotensive shock nonresponsive to fluid bolus and dopamine infusion and succumbed to the illness. His enzyme assay revealed normal beta-glucosidase level for Gaucher disease and a low level of sphingomyelinase, consistent with NPD.

His older sibling had similar features and was admitted in PGE ward at 12 years of age with periorbital puffiness around 8 months earlier and had expired in hospital. She had wasting and stunting, hypertension, encephalopathy, seizures, and shock. Ultrasonography of abdomen showed liver of 21 cm, normal echotexture, normal outline, and a distended gall bladder, spleen of 22 cm, normal echotexture, and normal kidneys. Echocardiography revealed mitral valve mildly thickened with prolapse of postmitral leaflet, moderate mitral regurgitation, and eccentric jet anteriorly directed and normal left ventricular ejection fraction. The parents were tested by Sanger sequencing of* SMPD1* gene and were heterozygous for the identified variant in child and were counseled accordingly for future pregnancies.

In* family 4*, a female child was brought with regression of milestones from 2 1/2-year age. She had history of neonatal cholestasis. At 4-year age she presented with fever and respiratory complaints. Examination revealed microcephaly, growth failure, hepatosplenomegaly, and upward gaze restriction. Fundus examination did not show any cherry red spot, and X-rays did not reveal any dysostosis. Keeping a possibility of Niemann-Pick disease, a sphingomyelinase assay was done, which showed normal sphingomyelinase enzyme in the blood lymphocytes. However, further testing by targeted DNA analysis was done for storage disorders by next generation sequencing (NGS) and two variants in* NPC1 *gene were identified. One was an exon 3 variant c.275 A>G (pGln92Arg), and an exon 22 variant c.3246 T>A (p.Ser1082Arg) confirming diagnosis of NPD type C. The family was counseled for subsequent prenatal diagnosis.

*Family 5.* A 9-month male child presented with developmental delay, respiratory problems, failure to gain weight. He was born full term by LSCS (indication:oligohydramnios) with birth weight of 2.2 kg. He had jaundice on day 2 of life requiring phototherapy for 3 days. From 4 months age, he developed on and off cough without significant fever. Locally he was admitted for 2 months and given antibiotics and oxygen. For positive CMV IgM, he received also ganciclovir for 2 weeks. Thereafter, he developed increasing respiratory distress and perioral cyanosis. Examination revealed length/height of 56 cm (-0.85 Z score) and head circumference of 37.5 cm (-1.83 Z score) and hepatosplenomegaly. The liver was 4 cm below RCM and 5 cm below the xiphoid; spleen was 4 cm below LCM with notch present. He had hypotonia with scarf sign crossing midline. He had anemia during hospital stay; but X-rays did not show any dysostosis. Chest X-ray was suggestive of interstitial pneumonia and CECT chest revealed multifocal areas of patchy consolidation with ground glass opacity and reduced volume of thymus. The transaminases levels were 191 and 133 U/L with normal serum creatinine. Stool for fat globules were 30-40/HPF, and flow cytometry for chronic granulomatous disease (CGD) was negative. Keeping a clinical diagnosis of Niemann-Pick disease, enzyme analysis was performed which showed normal sphingomyelinase level-1.8 units (N-1.8-8.5 nmol/hr/mg protein). However chitotriosidase was elevated (2041 units) above normal values (28.7-63 nmol/ml/hr) and bone marrow showed scattered histiocytes with eccentric nuclei and multivacuolated cytoplasm, suggestive of non-Gaucher storage disorder. Thus, NPC was not ruled out. Vit D level was low and he was given replacement therapy. Further DNA testing was done by targeted NGS and identified homozygosity for an insertion variant in* NPC2 *gene c.82 +2 ->G, which was found to affect splicing on bioinformatics analysis. Genetic counseling was done and feasibility of prenatal diagnosis was discussed.

## 3. Discussion

Mucopolysaccharidosis disorders and Gaucher disease are commoner lysosomal storage disorders (LSDs). Recently sphingolipidoses have been reported to be common metabolic disorders from Saudi Arabia [12]. Early diagnosis and early initiation of treatment, including enzyme replacement therapy (ERT) in selected disorders, can enable the affected children to have near normal life. Because of the variable clinical presentation and limited availability of diagnostic testing, these disorders are missed out during routine evaluation. In Niemann-Pick disease (NPD), the incidence for carrier of the gene-SMPD1, causing ASMD, is 1:120 in the general population and increases to 1:60 for individuals of Ashkenazi Jewish descent [13]. NPD type A disease manifests with persistent early jaundice, enlarging abdomen, hepatosplenomegaly, and poor developmental progress and failure to thrive [10]. Death usually occurs by 3 years of age.

In a Nepalese boy, 13-year-old presenting with gait abnormalities and supranuclear gaze palsy, bone marrow revealed abundant foamy cytoplasm suggestive of storage disorder. Mutation analysis revealed two variants in the* NPC1* gene c.302T>G F101C in exon 4 and another IVS 24+1G>A mutation [14]. This confirmed the diagnosis of NPC. NPD type C often present with prolonged neonatal jaundice may remain normal for 1-2 years ultimately leading to slowly progressive and variable neurodegenerative course. Hepatosplenomegaly in type C is less severe than type A or B and they may survive into adulthood. The underlying biochemical defect in type C is abnormal cholesterol transport which leads to sphingomyelin and cholesterol accumulation in the lysosomes and a secondary partial reduction in acid sphingomyelinase activity.* NPC1* and* NPC2 *are the genes underlying NPD type C. Few cases of NPC are reported compared to NPD [15–17]. In present study, we found one case with* NPC1*, with a likely deleterious variant in homozygous state. She had hepatosplenomegaly and gaze palsy and presented at 4 years of age. A high index of suspicion is needed for the diagnosis. Sometimes it is difficult to do filipin staining in hepatic cells by biopsy, but increased level of chitotriosidase as a surrogate marker helps in giving a clue to underlying diagnosis. Chitotriosidase is more elevated especially in ASMD cases than in NPC cases. Mutation studies are now available for NPC and can be done in individual cases. However, if VUS is identified, pathogenicity confirmation can only be done when another similar case is reported in another family. In the male infant with respiratory problems, we found an insertion mutation in the* NPC2* gene. Lung involvement is a dominant feature in NPD, and this may also lead to persistent oxygen requirement.

Small deletions or nonsense mutations in the* SMPD1* gene usually lead to NPD type A, whereas missense mutations that produce a defective enzyme cause a milder NPD type B phenotype [15, 18] ]. Levran et al. identified single base deletion in Ashkenazi Jews causing the pro330FS mutation, causing a frameshift leading to premature chain termination [7]. Most (65%) SMPD1 mutations are accounted for by three mutations, R496L, L302P, and pro330FS mutation in Ashkenazi Jewish NPD cases.

In the family 2, we found a 4 bp insertion variation (C>CCTGG) in exon 2 leading to a premature stop codon. This mutation is 35 amino acids from the amino acid position 355 in the* SMPD1* gene. On database analysis this mutation was predicted to disrupt protein function. Family 3 also showed novel missense variant c.1566T>G in exon 6 of the* SMPD1 *gene identified on NGS. This was predicted to be deleterious on analysis in available mutation specific databases, and not reported in general population.

In family 3, the siblings showed growth retardation, hypertension, and mitral valve prolapse in addition to hepatosplenomegaly. Cardiomyopathy and MVP are commonly reported in mucopolysaccharidoses but can also be seen in NPD. Mutation analysis also showed novel missense mutation in this family in exon 6 of* SMPD1* gene.

Various treatment modalities tried with no or little success are orthotopic liver transplantation in type A and cord blood transplantation in several type B. A phase I trial of enzyme replacement therapy for type B has been completed. Clinical trials of the drug Miglustat (substrate reduction therapy, SRT) have been performed and the drug has been approved in Europe for the treatment of type C disease [6]. SRT has emerged as promising form of treatment as single therapy or in addition to ERT for ASMD. Olipudase alfa is the recombinant enzyme, the human acid sphingomyelinase which has been found beneficial in treatment of ASMD [19]. There are only mild adverse events like nausea, abdominal pain, and headache, and no severe reactions were reported.

Chitotriosidase is a surrogate marker for diagnosis of Gaucher and Niemann-Pick disease and is significantly increased in these disorders. However, 6% of the population is deficient for chitotriosidase protein. In case the enzyme levels are inconclusive, the further mutation analysis can give a confirmatory diagnosis. New biomarkers are being developed to make diagnosis easier. One of these is plasma lysosphingomyelin which can of potential use in diagnosis of Niemann-Pick type B and also NPC [20, 21]. Recently, bile acid B has also been found to detect* NPC1* related disease on newborn dried blood spots [22]. Chitotriosidase and lysosphingomyelin levels have also been found to be helpful in monitoring response to therapy [19]. Recent research is also on to find new therapeutic options for NPC, and one of the studied molecules is 6-*O*-*α*-maltosyl-*β*-cyclodextrin (G2-*β*-CD) in treatment of* NPC1* disease [23]. Promising results have been demonstrated in* NPC1* deficient mice models.

In countries like India and nearby South Asian countries with low GDP, the numbers of diagnosed cases actually represent a minority of the actual number of cases in view of the large population base. There is discrepancy in resources and availability of diagnosis and treatment facilities for rare disorders [2, 3]. NPD also present with nonimmune hydrops fetalis (NIHF) and should be considered in pregnancies with recurrent hydrops. The availability of enzyme analysis and mutation analysis has made early prenatal diagnosis of NPD possible in selected families at risk of child with severe NPD. There is also a need to increase awareness regarding early diagnosis of these disorders. Till the treatment of NPD is made widely available, the focus lies on timely detection of index child with NPD and provision of genetic counseling and planned prenatal diagnosis in some families.

## Additional Points

*Key Message*. Niemann-Pick disease can have variable clinical presentations and suspicion is the key to early diagnosis. High incidence is expected in South East Asia region. A complete evaluation including DNA analysis can help in reaching an appropriate diagnosis.
